# Progress on Electrochemical Biomimetic Nanosensors for the Detection and Monitoring of Mycotoxins and Pesticides

**DOI:** 10.3390/toxins16060244

**Published:** 2024-05-26

**Authors:** Kavitha Lakavath, Chandan Kafley, Anjana Sajeevan, Soumyajit Jana, Jean Louis Marty, Yugender Goud Kotagiri

**Affiliations:** 1 Department of Chemistry, Indian Institute of Technology Palakkad, Palakkad 678 557, Kerala, India; 202304002@smail.iitpkd.ac.in (K.L.); 202105002@smail.iitpkd.ac.in (C.K.); 202314001@smail.iitpkd.ac.in (A.S.); 202205012@smail.iitpkd.ac.in (S.J.); 2 BAE Laboratory, Université de Perpignan Via Domitia, 52 Avenue Paul Alduy, 66860 Perpignan, France

**Keywords:** affinity-based sensors, biomimetic nanosensors, agricultural toxins, aptasensors, immunosensors, molecularly imprinted polymers, electrochemical sensors, mycotoxins, pesticides, herbicides

## Abstract

Monitoring agricultural toxins such as mycotoxins is crucial for a healthy society. High concentrations of these toxins lead to the cause of several chronic diseases; therefore, developing analytical systems for detecting/monitoring agricultural toxins is essential. These toxins are found in crops such as vegetables, fruits, food, and beverage products. Currently, screening of these toxins is mostly performed with sophisticated instrumentation such as chromatography and spectroscopy techniques. However, these techniques are very expensive and require extensive maintenance, and their availability is limited to metro cities only. Alternatively, electrochemical biomimetic sensing methodologies have progressed hugely during the last decade due to their unique advantages like point-of-care sensing, miniaturized instrumentations, and mobile/personalized monitoring systems. Specifically, affinity-based sensing strategies including immunosensors, aptasensors, and molecular imprinted polymers offer tremendous sensitivity, selectivity, and stability to the sensing system. The current review discusses the principal mechanisms and the recent developments in affinity-based sensing methodologies for the detection and continuous monitoring of mycotoxins and pesticides. The core discussion has mainly focused on the fabrication protocols, advantages, and disadvantages of affinity-based sensing systems and different exploited electrochemical transduction techniques.

## 1. Introduction

Mycotoxins, primarily created by biological molds, are dangerous pollutants in food and agriculture. They are highly resistant and may persist in foods and commodities at low levels, providing considerable harm to humans and animals because of their teratogenic, mutagenic, immunosuppressive, and carcinogenic qualities [1,2,3]. Aflatoxin B1 and M1 (AFB1 and AFM1), the most toxic mycotoxins, are classified as group 1 and group 2B carcinogens, respectively, by the International Agency for Research on Cancer (IARC), while ochratoxin A (OTA) and fumonisin B_1_ (FuB1) are categorized as group 2B human carcinogens [4]. The global mycotoxin issue has been addressed, with regulatory limits for mycotoxin residue in food, feed, and samples established by the European Union. For instance, the maximum allowable concentration of mycotoxins OAT should not surpass 2 µg/kg in wines and coffee, and 5 µg/kg in cereals. On the other hand, the acceptable range for AFB1 in various food items was decided to be between 0.05 and 20 µg/kg [5,6,7]. Therefore, developing sensitive and reliable mycotoxin detection technologies in different matrices is crucial to meeting worldwide needs.

Pesticides, referred to as plant protection products, used to manage pests, weeds, and diseases are among the most potentially hazardous and stable compounds introduced into the environment from synthetic sources [8,9,10,11]. Pesticides are characterized by chemical ingredients such as organochlorine (OC), organophosphate, carbamate, synthetic pyrethroids, etc. [12]. Pesticides can be categorized by their target, such as insecticides, nematicides, fungicides, weedicides, and others [13]. Various types of chemical compounds are used in agriculture and horticulture to control pests and diseases, promote plant growth, and manage unwanted vegetation. These compounds can be classified into different categories, including insecticides, molluscicides, fungicides, herbicides, and plant growth regulators [14,15,16]. Many developing countries, such as India, rely heavily on agriculture and pesticides. 

Mycotoxins and pesticides commonly affect agricultural crops such as vegetables, fruits, food, and beverage products [17,18]. Since the late 20th century, many nations have restricted the use of organic pollutants due to their high toxicity and extended half-lives, which are linked to several chronic disorders. Organic compounds are difficult to degrade naturally and remain present in the environment, transferring and bioaccumulating via the food chain [19,20,21]. To maintain high food production, pesticide use is almost inevitable. Governments have issued laws to regulate pesticide use and prescribe maximum residue levels for water and agricultural products. However, the use of pesticides that are excessive, inappropriate, and unlawful persists, particularly in developing nations [22,23].

The rapid, sensitive, selective, and consistent assessment of mycotoxins, pesticides, and residues is critical. Numerous approaches have been introduced, including high-performance liquid chromatography (HPLC), gas chromatography (GC), liquid chromatography-mass spectroscopy (LC-MS), enzyme-linked immunosorbent assays, and immunosensors [24]. However, their conventional methods, high expense, and need for training make them unsuitable for field operations. Sample preparation, such as for GC, is time-consuming, limiting the number of samples in a given timeframe [25,26,27,28,29]. Also, several pesticides disintegrate during chromatographic analysis at high column and injection head temperatures [30]. Since 1962, electrochemical sensors have been extensively studied in various fields, including medical, natural, and engineering sciences. They offer real-time monitoring due to their miniaturization, affordability, and simplicity. Electrochemical biosensors have emerged as an economical and portable alternative for pesticide detection, offering highly sensitive and selective determination with rapid response times and the potential for field testing [31,32,33,34,35,36].

Electrochemical biosensors can be integrated into bio-recognition molecules in the development of sensor design. The biosensor application domain’s most commonly employed bio-receptor elements include enzymes, antibodies, and aptamers [37]. Factors such as amino acid structural changes at the enzyme’s active region can significantly impact enzymatic activity, substrate selectivity, and stability of the sensing reaction. Enzyme denaturation is crucial to enzymatic biosensors. Variations in pH, pressure, ultraviolet exposure, temperature, organic solvents, detergents, or chemicals can cause enzyme denaturation [38]. Enzyme isolation and integration into an in vitro environment can reduce enzymatic activity. Immunoassays are a promising alternative to enzymatic tests due to their high-affinity interactions between antibodies and antigens, resulting in increased sensitivity and reduced detection limits [39]. The protein nature of antibodies makes them susceptible to denaturation under many experimental and physiological conditions. In addition, the inherent limitations of antibodies, such as animal or cell culture synthesis, complex preparation, low stability, high cost, and immunogenicity, limit the scope of their use. Biomimetic nanosensors, such as molecularly imprinted polymer (MIP)-based and aptamer-based sensors, are appealing for mycotoxin and pesticide detection due to their simplicity, sensitivity, accuracy, and reliability [39,40].

**Figure 1:**
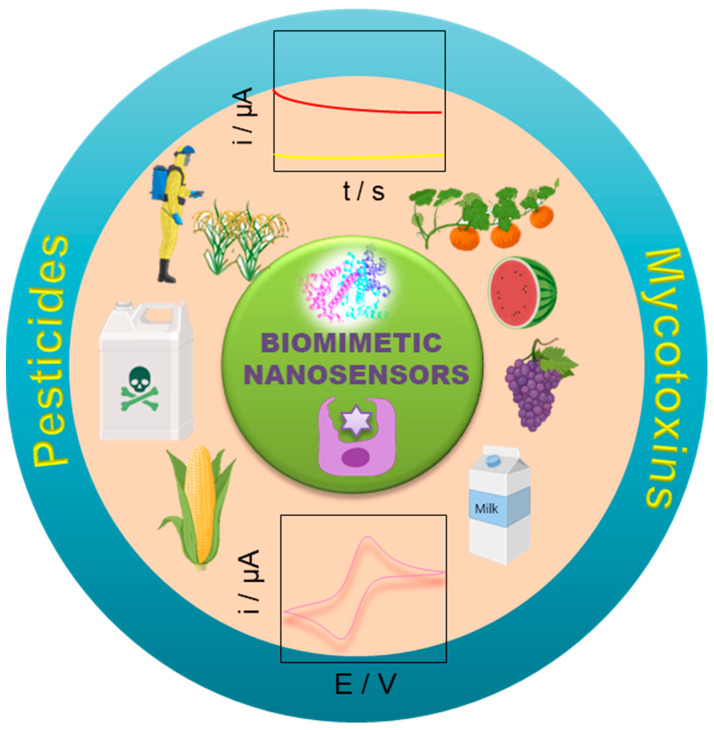
Schematic representation of an overview of the biomimetic nanosensors towards the detection of mycotoxins and pesticides.

**Figure 2:**
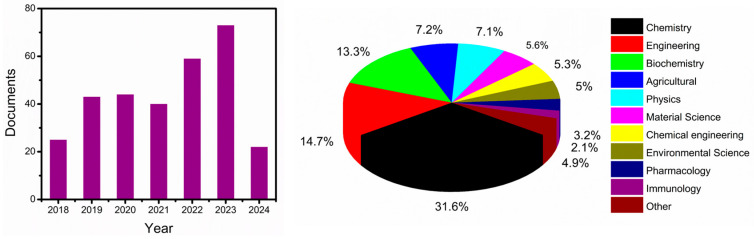
Scopus search results of mycotoxins and pesticide detection through electrochemical MIP/aptamer-based sensors for the period of 2018 to 2024.

Several reviews have been published in recent years related to the detection of mycotoxins and pesticides [41,42,43]. Most of these reviews mainly focus on immunosensors [44] and chemical-based sensors [17,41]. No review attempts have been made specifically on biomimetic nanosensors. This review article discusses recent advancements in affinity-based biomimetic sensors and assays, with an emphasis on aptamers and MIP as recognition elements. The overall view of the current review paper is represented in Figure 1 as a schematic illustration. We mainly focus on some of the important mycotoxins and pesticide detection that are extensively employed. It discusses the application of these bio-receptors in electrochemical methods for the continuous monitoring of agricultural toxins, such as mycotoxins, pesticides, and herbicide residues, in food and environmental samples. The article evaluates the advantages and disadvantages of different detection methods to find the most rational and sensitive design for mycotoxins and pesticide residue detection. It also discusses the principal mechanisms and recent developments in affinity-based sensing methodologies for continuous monitoring of agricultural toxins, focusing on the fabrication protocols, advantages and disadvantages of affinity-based sensing systems, and various electrochemical transduction methods. Moreover, we have cited the recent 5 years of literature related to biomimetic nanosensors for the detection of mycotoxins and pesticides. We searched the literature (2018 to till now) on the Scopus database with the keywords “mycotoxins, pesticides, electrochemical detection, MIP, and aptamer”. A total of 306 articles (223 articles, 72 reviews, 9 book chapters, and 2 conference proceedings) were found. The respective Scopus search results are displayed in Figure 2. 

## 2. Biomimetic Nanosensors for Mycotoxins

### 2.1. Nano-Electrochemical MIP-Based Sensors for Mycotoxins

#### 2.1.1. MIPs as Bioreceptors

**Figure 3:**
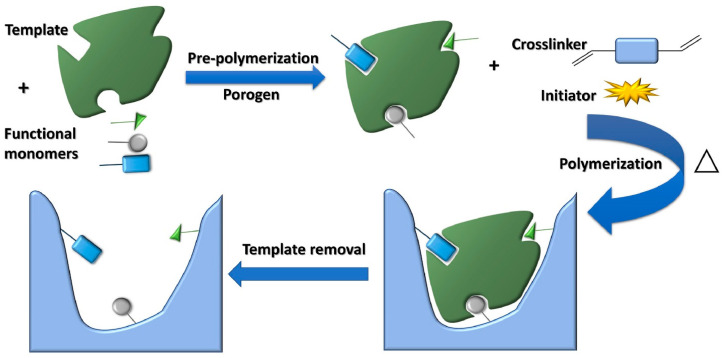
Schematic representation for the synthesis of MIPs. (i) Self-assembly of the template, functional monomer, and cross-linker. (ii) Polymerization of the template with a monomer and crosslinker. (iii) Removal of the template from the molecular imprint and creation of cavities. Reproduced with permission [50].

MIPs are versatile biomimetic molecular receptors that act as artificial antibodies. The fabrication of MIPs involves choosing the functional monomer, cross-linker, solvent, synthesis, and removal technique. As shown in Figure 3, MIP is made in a process that can be broken down into several steps: (i) The first step is for the analyte, functional monomer, and cross-linker to self-assemble; (ii) this is followed by the polymerization of the template with the monomer and the crosslinker; (iii) then, removal of the target analyte from the molecular imprint and creation of cavities occur. They are made by co-polymerizing a cross-linking and a complex formed between the target molecule and functional monomers, and the possible interactions among them are covalent, non-covalent, or semi-covalent interactions. There are three main categories of molecular imprinting techniques: bulk imprinting, surface imprinting, and epitope imprinting. After imprinting the surface, subsequent removal of the target analyte from molecular imprints occurs, and imprinting of cavities is conducted [45]. There are some widely used removal techniques, such as Soxhlet extraction, the microwave-assisted method, ultrasonication, electrochemical techniques, and proteolytic digestion [46]. The removal of the template molecule leaves behind specific voids known as imprints. These cavities correspond to the target molecule in size, shape, and chemical functionality [47]. The removal of the template and the rebinding of target analytes are the most important variables that contribute to the optimal functioning of MIPs. The binding efficiency of MIPs depends on the selectivity of the functional monomer, cross-linker, and analyte. Despite improved selectivity, MIP-based biosensors have limitations, including extended analysis durations, irregular diffusion of target molecules, and the uneven distribution of binding sites [46,48].

Polyakov and Dickey discovered imprints in porous silica particles in 1931 and 1949, respectively. Organic polymers were developed in the 1970s, with the noncovalent approach introduced in the 1980s. Various methods, including thermal heating, photopolymerization, microwave irradiation, sonochemistry, soft lithography, and electrochemistry, can facilitate the synthesis of micro-induced polymers [49]. 

**Figure 4:**
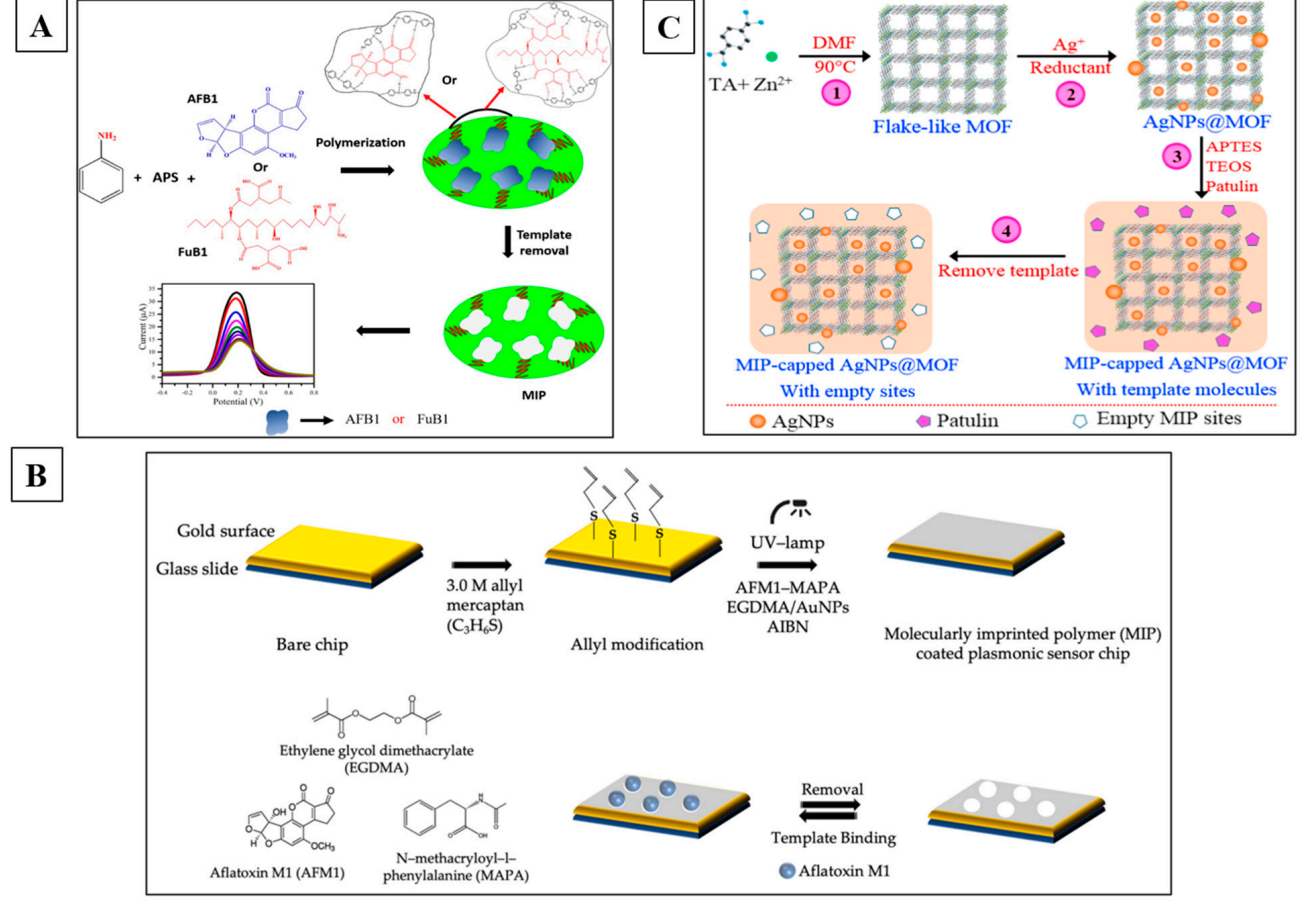
MIP-based electrochemical sensor for mycotoxins detection. (**A**) MIP-based techniques for the evaluation of the mycotoxins AFB1 and FuB1 [50]. (**B**) A nano MIP-based plasmonic sensor for AFM1 detection in raw milk samples [55]. (**C**) A novel MIP-capped AgNPs@ZnMOF sensor for patulin detection [58].

The sensor’s robust performance for the investigated analytes is expected due to the excellent conductivity of nanomaterials, along with the equivalent selectivity of MIPs [51]. As a result, widespread MIP-based electrochemical sensors have applications in the fields of agriculture and environmental toxic sensing. This section thoroughly discusses the recently reported MIP-based nanosensors for mycotoxin detection. Figure 4 represents the schematic illustrations of some of the important biomimetic nanosensors based on electrochemical transduction techniques. 

#### 2.1.2. AFB1 and FuB1

AFB1 is a poisonous substance that is generated by fungus and associated with several health hazards as well as environmental hazards [52]. This study presents the fabrication of a sensitive MIP-A/ITO and MIP-F/ITO-based electrochemical sensing platform developed to study the presence of AFB1 as well as FuB1. A simple chemical oxidative polymerization process was used to make the sensor platform from polyaniline (PANI) as an array and AFB1 and FuB1 as template molecules. These sensors offer excellent sensitivity, reliability, and ease of use [50]. And, some other groups developed MOF-DES/MIPs-based sensors for AFB1 detection from a cereal sample [53].

#### 2.1.3. AFM1

Aflatoxins, a family of hazardous and cancer-causing substances, pollute agricultural products. When dairy cows consume contaminated diets, the feeds are digested and changed into a primary kind of AFM1, which is a cancerous type of aflatoxin. This major form of aflatoxin is removed through milk. The identification of AFM1 in milk is essential for ensuring the purity and hygiene of food [54]. A platform for recognizing the presence of aflatoxins has emerged in recent years, and it is comprised of plasmonic sensors that have MIPs. In order to detect a small quantity of AFM1 in milk, a MIP-based plasmonic sensor was developed employing gold nanoparticles (AuNPs) embedded into polymer nanofilm and a sensor that had been coated with allyl mercaptan. In addition to having a limit of detection (LOD) of 0.4 pg/mL, the MIP-based sensor exhibited a broad linear range that extended from 0.0003 ng/mL to 20.0 ng/mL. This unique and extremely sensitive surface plasmon resonance-detecting platform might be used as an alternative strategy for milk quality control because it exhibits ease of use, rapidity, affordability, and outstanding selectivity and specificity [55]. 

A recent study on AFM1 sensing demonstrated an excellent level of sensing at the picogram scale, with an LOD of 0.322 pg/mL. The PANI-based MIP sensors achieved a high level of sensitivity of 7.037 µA mL/ng cm^2^, reliability, and specificity towards interferents using a PANI film which was imprinted on ITO-coated glass substrate using an oxidative polymerization process. PANI is bound to the template molecule, AFM1, via intermolecular interactions like hydrogen bonds, which are transient in existence and can be removed very easily in the MIP network, making the sensing more robust [41]. 

In 2017, Rana Shadjou and colleagues demonstrated another technique for detecting AFM1. A multilayer film was formed by electrodepositing silver nanoparticles (AgNPs) into a nanocomposite consisting of α cyclodextrin as the conductive matrix and graphene quantum dots (QDs). This film was then coated on a glassy carbon electrode (GCE), resulting in a sensing platform with a linear range of 0.015 mM to 25 mM and an extremely low LOD of 2 µM. Furthermore, they effectively demonstrated its practical feasibility by quantifying AFM1 in raw milk samples [56].

#### 2.1.4. Patulin

Patulin is a common mycotoxin found in fruits, cheese, and other musty foods, and its high solubility in water can cause various side effects. The World Health Organization (WHO) and the European Union have set limits on the maximum daily level of patulin in food and agriculture products, with the WHO restricting it to 0.4 µg/kg body weight and the European Union setting it at 10 μg/kg [57]. This study presents a novel method for selectively determining patulin, employing AgNPs in a zinc metal–organic framework (AgNPs@ZnMOF). To create a selective interaction with patulin molecules, an MIP matrix is created on the AgNPs@ZnMOF by using the surface-imprinting technique. The combination of the MIP’s specifically detecting property and the peroxidase-like mechanism of the unique AgNPs@ZnMOF nano compound encouraged the synthesis of a sustainable sensor for patulin detection. The MIP-capped AgNPs@ZnMOF that was synthesized was able to catalyze the reaction between hydrogen peroxide (H_2_O_2_) and terephthalic acid, resulting in the production of a high fluorescent compound that possessed an LOD of 0.06 µmol/L. Patulin could be measured in complicated media using this approach, and there was no substantial interference from analog chemicals [58]. In other work, a novel sensor was developed for the measurement of patulin, utilizing the square wave voltammetry (SWV) approach. This sensor employed a GCE that was modified by a composite of ionic liquid-based MIP and magnetic nanoparticles/graphene oxide (Fe_3_O_4_/GO). The high conductivity of GO nanosheets enhanced the sensitivity of the sensor, while the presence of specific cavities for patulin molecules in MIP led to an enhancement in sensor selectivity. The sensor demonstrated a remarkable linear range spanning from 0.001 nM to 250.0 nM, with a quantification limit of 0.001 nM and an LOD of 3.33 × 10^−4^ nM. In addition, the sensor was successfully employed to quantify the level of patulin in a real sample of apple juice [42]. 

An electropolymerization process was used to develop a film of poly (thionine) coated with platinum nanoparticles (PtNPs) over a prepared surface of PtNP-nitrogen-doped graphene (NGE) with a thionine tail. This film exhibited a high capacity and fast kinetics for uptaking patulin molecules. The utilization of the MIP film and thionine-PtNP-NGE in double amplification was confirmed to enhance the detection of patulin with high sensitivity and selectivity. Thus, the designed sensor showed exceptional performance in detecting patulin within the range of 0.002–2 ng/mL, with an LOD of 0.001 ng/mL. Furthermore, the sensor also exhibited superior stability, as well as repeatability and selectivity [59]. 

#### 2.1.5. Ochratoxin A and B

OTA is a mycotoxin produced by Aspergillus and Penicillium fungi that structurally consists of a para-chlorophenols group containing a dihydroisocoumarin moiety. OTA, identified globally in many food items, can have several toxicological effects, such as nephrotoxicity, hepatotoxicity, neurotoxicity, teratogenicity, and immunotoxicity. These are synthesized during the storage of many plant-derived products, including cereals, cereal products, herbs, and spices. Several metabolites associated with OTA have been found, including ochratoxin B, which is the dechloro analog of OTA, and ochratoxin C, which is its ethyl ester. OTA is one of the most commonly found mycotoxin pollutants in human blood samples from the European regions. OTA is not stored or deposited in the body, but its uneven distribution throughout the body can cause significant harm to the kidneys. The toxin was categorized as a 2B carcinogenic chemical, indicating its potential to cause cancer in people [60].

In another study, a voltammetric sensor was developed to measure OTA in food samples. This was achieved using cyclic voltammetry (CV) electropolymerization, where a polypyrrole film was applied onto the surface of a GCE modified with multi-walled carbon nanotubes (MWCNT). The polypyrrole film exhibited selective binding to OTA molecules and enhanced selectivity with an LOD of 1.7 μg/L. The MIP sensor exhibited stability, ease of operation, and repeatability in its response. It has been employed for the analysis of OTA in beer and wine samples as a real sample analysis [43].

Xiaopeng Hu and colleagues published a recent study where they developed a ratiometric electrochemical sensor using MIP. This sensor had excellent selectivity and reproducibility for detecting OTA. The MIP was synthesized using CV, and a magnetic field was applied during electropolymerization to control the orientation of the MIP, thereby enhancing the efficiency of molecular recognition. The substrate employed for signal amplification and the reference signal were a mix of AuNPs, poly (ionic liquid) and flavin mononucleotide-decorated carbon nanotubes—MoS2 nanosheets (AuNPs/PIL-FMNS/CNT-MoS2). The OTA quantification was determined by calculating the ratio of the peak currents, which exhibited a linear relationship with the concentration of OTA. The results revealed an excellent sensing platform, with a linear response ranging from 0.5 to 15 μM and a low LOD of 14 nM [61]. 

#### 2.1.6. Trichothecenes

Trichothecene mycotoxins are frequently present in many foods and crops, such as oats, wheat, corn, beans, and rice. These toxins primarily originate from fungi belonging to taxonomical taxa. Fusarium mold is a highly lethal plant pathogen that falls under the taxonomical genus of fungi. The production of trichothecene mycotoxins, such as T2-HT2 toxins and deoxynivalenol (DON), falls under the categories of type-A and type-B trichothecene. Type A is known to be highly toxic and can lead to severe health problems, such as skin, liver, and kidney disorders, due to their enhanced ability to be absorbed via the skin. The European Food Safety Authority has established a maximum acceptable daily intake (TDI) limit of 0.1 µg/kg body weight/day for T2-TH2 toxins [62]. Recently, researchers have created sensors based on MIP-Fe^3+^/GCE for detecting T2 in various food items (such as corn, rice, and soybean) and human serum. These sensors have a broad detection range, from 1.12 nM to 2.12 mM, and an LOD of around 0.15 ng/g [63].

A new biosensor was constructed using DNA aptamers, where rGO-TEPA-Au@Pt NRs were immobilized with a signal DNA probe. Entrapment of T-2 toxin results in a modification of the signal. The signal was significantly amplified through the catalytic activity of H_2_O_2_ and quantified using chronoamperometry. The amplified response was outstanding, yielding an LOD of 1.79 fg/mL (3 standard deviations above the mean) and a wide linear range spanning from 10 fg/mL to 100 fg/mL [64].

A food sample (such as oats, wheat, corn, etc.) was contaminated and poorly stored, resulting in the presence of type-B trichothecene mycotoxins, specifically DON and its acetyl derivative. The UN Food Council and the European Union have established a maximum TDI limit of 1.0 µg/kg body weight/day [62]. A new finding involves the use of F-MWCNTs combined with MIP L-Arginine to detect DON using the CV method. The sensor that was created has a broad range of LOD, spanning from 0.1 to 70 μM, with an LOD of 0.07 μM when applied to wheat as a real sample [65]. PtPd nanoparticles/PEI-rGO have been utilized in recent years to assist in the detection of DON using an Exonuclease III aptamer-based sensor. The sensor has a detection range of 1 × 10^−8^ mg/mL to 1 × 10^−4^ mg/mL, with an LOD of 6.9 × 10^−9^ mg/mL in maize flour samples [66]. Table 1 summarizes the recent literature on MIP-based electrochemical sensors for the detection of mycotoxins and pesticides. 

### 2.2. Electrochemical Aptamer-Based Sensor for the Detection of Mycotoxins

Aptasensors are one of the important biomimetic nanosensors that have unique advantages like selective and sensitive detection, chemically synthesizable, and stability in different environmental conditions. Moreover, we can modify the electrochemical optical labels over one end of the aptamer sequence and use it as an indirect recognition matrix. In this current section, we describe the recent development of electrochemical label and label-free apatasensors for the detection of mycotoxins. Table 2 summarizes the recent reports of the electrochemical aptasensors for the detection and continuous monitoring of mycotoxins.

#### 2.2.1. Labelled Aptasensors

Label sensory mechanism: Labels are commonly used in bio-recognition detection systems across various fields. Label molecules can range from radioactive or fluorescent dyes to metal complexes or nanoparticles [90,91]. A label is typically added to the target molecules or bioreceptors. The analysis involves evaluating label activity or changes in chemical or physical characteristics on the transducer surface. Label-dependent technology has benefits, but labeling and immobilization are time-consuming and costly. Moreover, altering binding characteristics can lower biosensor repeatability, sensitivity, and selectivity [92].

The exceptional qualities of electrochemical detection, such as its affordability, simplicity, sensitivity, and quick reaction, have gained a lot of interest in recent years. Electrochemical aptasensors are capable of producing electrical signal responses resulting from the electron transfer influenced by the particular interaction between aptamers and target mycotoxins when the aptamers are introduced on an electricity-conducting substrate, such as an electrode made of gold or carbon. To analyze mycotoxins in food and agricultural products, several electrochemical aptasensors, such as potentiometry, linear sweep voltammetry, SWV, field effect transistor, CV, and differential pulse voltammetry (DPV), were developed [3,93,94]. Researchers are very interested in electrochemical aptasensors because of their high sensitivity, selectivity, and efficiency, as well as their advantages of being quick, portable, and inexpensive.

Regarding the electrochemical aptasensor for trace mycotoxin detection, sensitivity is a significant concern despite its great selectivity and adaptability. Amplification of the electrochemical response is essential for achieving high sensitivity. However, the interaction of a tiny molecule such as mycotoxin with its specific aptamer does not noticeably affect the charge-carrier density. Consequently, altering the electrode surface offers a crucial way to enhance electrochemical performance and achieve signal amplification. Functional nanomaterials, such as organic and inorganic nanoparticles with exceptional qualities, including a large specific surface area, ease of functionalization, and strong biocompatibility, are incorporated into the sensing platforms to enhance the sensing performance of electrochemical aptasensors.

Firstly, Wang et al. [84] successfully used an electro-chemiluminescent aptasensor approach for OTA measurement in wheat samples. This method was based on the AuNPs-modified electrode and luminescence-labeled particular aptamer. The electro-chemiluminescent aptamer biosensor was created by immobilizing the complementary DNA sequence of the aptamer on the surface of an electrode that had been treated with AuNPs. The DNA 2 sequence hybridized with the ABEI-labeled aptamer, which was then used as an electro-chemiluminescent probe. When the aptamer recognized the target OTA, it produced a diminished electrochemiluminescence (ECL) signal. This caused DNA 2 (the aptamer electro-chemiluminescent probe labeled with ABEI) to separate from DNA 1 and travel further away from the electrode surface. The ideal conditions showed a drop in ECL intensity proportional to OTA concentrations from 0.02 to 3.0 ng/mL, with an LOD of 0.007 ng/mL.

Typically, redox agents such as methylene blue (MB), ferrocene (Fc), and toluidine blue are added to increase the electron transfer efficiency even further. The distance-dependent current responses produced by a terminal redox-labeled probe can be directly impacted by the target-induced conformational change in the aptamer. Therefore, Goud et al. [20] created a functionalized GO-based aptasensor for AFB1 detection, employing a blue-labeled methylene aptamer as the signaling fragment. In addition to acting as a signal-enlarging platform by enhancing the conductivity and catalytic qualities of the sensor, the functionalized GO was deposited onto the surface of screen-printed carbon electrode (SPCE) to bridge the aptamers and the electrode. Current signals increased as a result of the AFB1-triggered conformational shift in MB-tagged aptamers, which brought MB and the electrode closer together. AFB1 may be detected by the aptasensor at as low as 0.05 ng/mL along a linear range of 0.05–6.0 ng/mL in milk and serum samples.

Wang and coworkers created MB-labelled electrochemical aptasensors in 2020. Variations in electrode distance and interactions with aptamer bases during affinity binding can impact the current signal. The electrochemical aptasensors were created with sensitive and substantial responses to targets by screening various sites and attaching MB tags to specific sites. Using a 26-mer DNA aptamer with MB on an internal T site and a thiol moiety at the 5′ terminals, the group created an electrochemical aptasensor sensor on the gold electrode for rapid and sensitive AFB1 detection. This sensor detected AFB1 in wine, maize flour, and milk samples with an LOD of 6 pM and exhibited outstanding signal-on responses [95].

Zhu et al. created a dual-ratiometric electrochemical aptasensing method to detect AFB1 and OTA. To build the Fc-labeled AFB1 aptamer and MB-labeled OTA aptamer, different binding sites were created using AQ-labeled complementary DNA (cDNA). Target-induced current ratios (IFc/IAQ and IMB/IAQ) were employed to quantify the relationship between AFB1 and OTA. To compare performance, two types of aptasensors were created using hairpin DNA (hDNA) and linear single-stranded DNA (ssDNA) as the cDNA. The study found that stiff 2D hDNA enhances sensing interface construction and recognition efficiency, resulting in a highly sensitive, reliable, and anti-interference aptasensor. The hDNA-based aptasensor detected AFB1 at 10–3000 pg/mL and OTA at 30–10,000 pg/mL without cross-reactivity. Additionally, the aptasensor was tested on maize and wheat samples, and HPLC-MS/MS confirmed its reliability [96].

A simultaneous electrochemical aptasensor was designed by Dehaghani and his group to detect OTA and AFB1. Hemin@HKUST-1 and Fc@HKUST-1 were synthesized by encapsulating hemin and Fc in HKUST-1 MOF, forming from copper nodes and trimesic acid. These molecules bind to complementary DNA sequences of OTA (cDNA1) and AFB1 (cDNA2), respectively. Next, the hemin@HKUST-1/cDNA1 and ferrocene@HKUST-1/cDNA2 bioconjugates were deposited over a GCE embellished with AuNPs-CNDs. The bioconjugates’ current response to hemin and Fc electroactive labels were evaluated at two potentials simultaneously using DPV in the absence of mycotoxins. The presented aptasensor can quantify OTA and AFB1 mycotoxins from 1.0 × 10^−2^ to 100.0 ng/mL. Additionally, OTA and AFB1 had LODs of 4.3 × 10^−3^ ng/mL and 5.2 × 10^−3^ ng/mL, respectively [97].

**Figure 5:**
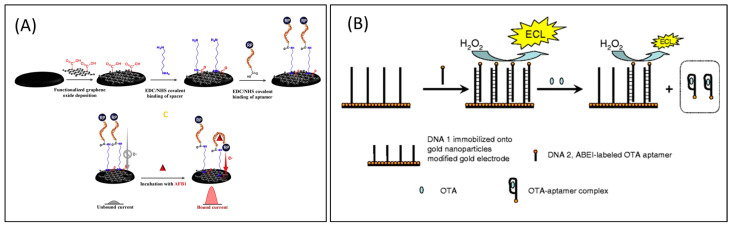
Electrochemically labeled aptasensors for the detection of mycotoxins. (**A**) Functionalized GO-based electrochemically labeled aptasensor for AFB1 detection employing an MB-labeled aptamer as the signaling fragment [20]. (**B**) AuNP-modified gold electrode and luminescence-labelled aptamer approach for OTA measurement in wheat samples [84].

Nguyen et al. [85] reported an electrochemical aptasensor for AFM1 determination based on this particular aptamer. This aptasensor integrated the signal enhancement function of Fe_3_O_4_ magnetic nanomaterials with the distinct identification capacity of aptamers. Within the detection range of 6–60 ng/L, an LOD of 1.98 ng/L was achieved under the conditions of this amplification process. However, employing the same particular aptamer, an electrochemical impedance biosensor was reported for AFM1 determination, and the proof of this technique in milk samples was examined. Figure 5 represents the schematic illustrations of some of the important electrochemical-labeled aptasensors for the detection of mycotoxins.

#### 2.2.2. Label Free Aptasensors

**Figure 6:**
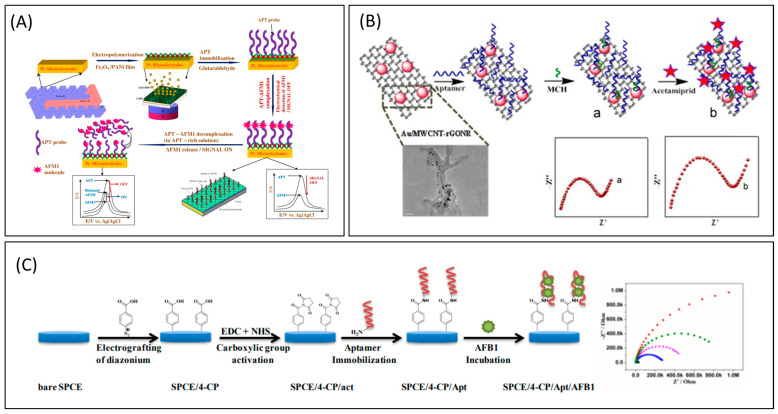
Electrochemical label-free aptasensors for the detection of mycotoxins. (**A**) Principle of label-free detection of AFM1 with magneto-electrochemical Fe_3_O_4_/PANi-based aptasensor [85]. (**B**) Au/MWCNT-rGONR based label-free electrochemical aptasensors for the detection of acetamiprid [103]. (**C**) Label-free electrochemical aptasensor, SPCE/4-CP/Apt/AFB1 for the detection of AFB1 [94].

Label-free sensory mechanism: The label-free sensing mechanism eliminates the need for labeling procedures. Neither the target molecule nor the bioreceptors undergo any modifications. Instead, they are utilized in their unaltered state. The bioreceptor is fixed onto the transducer surface, and the sample containing the desired substance is immediately exposed to the modified surface. Analyzing the surface involves evaluating the alteration in electrical or physical characteristics that are influenced exclusively by the affinity of the interaction between the analyte and its receptor and, consequently, the concentration of the analyte in the sample. Label-free monitoring enhances the preservation of high affinity and reduces non-specific adsorptions [98]. Figure 6 represents the schematic illustrations of some of the important electrochemical-labeled free aptasensors for the detection of mycotoxins.

Direct interaction with the target mycotoxin causes aptamers on the electrode’s surface to undergo a conformational shift, affecting the electrical characteristics of the sensing platform. The target–aptamer complex’s steric hindrance affects electron transfer processes. On the contrary, the conformational transition of aptamers impacts the current response of electroactive substances. Based on this concept, KY Goud and his team [94] developed label-free electrochemical aptasensors to detect AFB1 sensitively and selectively. According to research, AFB1 is extremely carcinogenic and mutagenic. It is crucial to monitor AFB1 pollution to minimize health risks. A label-free electrochemical impedimetric aptasensor for AFB1 detection was developed. The research group examined the analytical performance of two aptamer sequences (seqA and seqB). An aptamer covalently bonded as a compact monolayer on SPCEs was used for detection through a diazonium coupling reaction. AFB1 was quantified using electrochemical impedance spectroscopy (EIS). Both types of aptamer sequences yielded a dynamic quantification range of 0.125 to 16 ng/mL, with LODs of 0.12 and 0.25 ng/mL for seqA and seqB, respectively. The aptasensors were tested in beer and wine samples, achieving 92–102% recovery rates for AFB1 detection.

Nanomaterials with high conductivity are popular substrates for electrode decoration. Nanoscale inorganic materials can change electrode surfaces and carry bioprobes for different electrochemical aptasensors. AuNPs are widely employed in electrochemical aptasensors due to their high conductivity, variable sizes, facile production, and easy conjugation with biomolecules [99]. An electrochemical aptasensor for OTA measurement was developed using a gold electrode and aptamer identification. Evtugyn and his coworker created an electrochemical aptasensor for OTA detection using a gold electrode and electropolymerized AgNPs and neutral red produced through chemical reduction with catechol-containing macrocyclic ligands. The Ag-S bonding covalently linked thiolated aptamers against OTA to AgNPs. The aptamer’s conformational transition caused by OTA contact increased the charge transfer resistance, as evaluated by using EIS in the presence of ferricyanide ions. Later, the electrochemical aptasensors were developed and used to detect OTA from beer samples, with satisfactory reviews [82].

Another example is using AuNPs to create label-free electrochemical aptasensors for FuB1 detection using polydimethylsiloxane (PDMS) film-based micro-cells and SPCE. This approach involves covering the pretreated SPCE with a hole-filled PDMS sheet to create a micro-cell with three electrodes at the bottom. Highly distributed AuNPs were electrodeposited on working electrodes to act as thiolated aptamer immobilization matrices. A single nanoparticle can be coupled with many aptamer probes, and the dense covering of AuNPs on SPCE provided numerous binding sites for aptamer binding, significantly boosting the impedance signal. The proposed aptasensor demonstrated good linearity from 10 to 50 ng/mL, with a low LOD of 3.4 pg/mL (S/N = 3). The designed aptasensing apparatus is cost-effective, simple, sensitive, low-reagent consumption, and adaptable to various mycotoxins with available aptamers [99].

Another study shows the use of AuNPs for the development of an electrochemical aptasensor for detecting AFB1 by depositing AuNPs on a GCE modified with zeolitic imidazolate framework-8 (ZIF-8). The greater surface area of the AuNPs/ZIF-8 nanocomposite led to higher aptamer loading on the electrode. Relative to existing sensors, the developed aptasensor had a larger linear range from 10.0 to 1.0 × 10^5^ pg/mL and a lower LOD of 1.82 pg/mL under optimized conditions. The results showed that the aptasensor had excellent selectivity, repeatability, and stability. The aptasensor also detected AFB1 in maize and peanut oil samples with good recoveries [100].

Not only were AuNPs used to develop sensitive aptasensors, as QDs were also employed for the sensitive detection of mycotoxins. An ultrasensitive label-free aptasensor was developed by Rahimi and his group [101] by using an AFB1 aptamer immobilized on carbon quantum dots/octahedral Cu_2_O nanocomposite. Electrochemical measurements such as EIS and DPV were employed. Taguchi’s approach resolved this issue by optimizing experimental settings with fewer experiments. Under ideal conditions, electrochemical signals decreased with increasing AFB1 concentrations, with a dynamic range of 3 ag/mL–1.9 µg/mL and a low LOD of 0.9 ± 0.04 ag/mL. The results showed satisfactory reproducibility, selectivity, stability, and reliability.

Li and his group [102] synthesized a new carbon dots–black phosphorus nanohybrid (CDs-BP), which was used to develop a new label-free electrochemical aptasensor for ultrasensitive detection of OTA. Nanocomposite morphology and structure were examined using advanced techniques, including transmission electron microscopy (TEM), X-ray photoelectron spectroscopy, and Zeta potential. Through C–P bonding, the CDs-BP nanohybrid effectively immobilized aptamers, improving electrochemical sensing efficacy for OTA detection. The aptasensor demonstrated a flexible linear range for OTA from 0.1 fg/mL to 10.0 ng/mL and a low LOD of 0.03 fg/mL under ideal conditions. The suggested aptasensor showed strong stability and OTA detection selectivity. Additionally, practical findings have been obtained for wheat and grape juice samples. 

## 3. Biomimetic Nanosensors for the Detection of Pesticides

Pesticides are chemical substances used to control pests. Their consumption worldwide is estimated at two million tons per year, with 45% in Europe, 25% in the USA, and 25% in other nations [104]. Without pesticides, one-third of agricultural production would be lost. Among various kinds of pesticides, OC pesticides have long been widely used for pest management. Later, they were replaced with less toxic and more effective organophosphorus (OPs) pesticides due to their hazardous consequences. OPs were formerly thought to be a safe alternative to OCs, but their widespread usage, accumulation, and exposure have caused severe toxicological consequences on organisms that are not targeted [105,106,107]. OP compound poisoning is a global health issue, causing over three million poisonings and 200 thousand fatalities yearly [13,108,109,110]. 

Diazinon, a widely used organophosphate pesticide, is categorized in group 2A by the IARC [111]. A unique and effective porous adsorbent nanoparticle (MIP@MOF) was utilized to quantify diazinon from the air by applying a needle trap device (NTD). The sorbent was characterized using various techniques, and the optimal temperature and humidity were determined. The method’s performance was evaluated for reliability using the NIOSH 5600 standard technique. Furthermore, the results showed acceptable accuracy and a significant correlation coefficient (R^2^ = 0.9781). The MIP@MOF:NTD strategy effectively monitors diazinon consumption in real-life conditions, demonstrating its potential as a solvent-free, quick, and environmentally friendly method [71]. Parathion methyl (MP) is another highly hazardous pesticide, and it is prohibited for use in the majority of countries. It can penetrate the body by way of breathing, through the absorption of the skin, or even by swallowing, and it can also enter the eyes. This substance can cause various symptoms, such as abdominal pain, nausea, and many more. The WHO has designated it as an IA, which stands for “extremely hazardous” [112]. The extensive improper utilization of MP constitutes a substantial risk to both the health of nature and human health. An electrochemical sensor was designed with a zr metal–organic framework-loaded curcumin (CCCM/UiO-66/GCE) to selectively detect MP. The integration of CCM’s distinctive electrochemical properties and UiO-66’s high porosity for retention improved the sensor’s sensitivity. The sensor demonstrated a broad linear range, exhibited a dynamic linear variation of 20 to 20,000 ng/mL, and had a low LOD of 0.98 ng/mL. Furthermore, the sensor also demonstrated quite good repeatability, durability, and sensitivity. The quantitative analysis of MP in agricultural samples can be accomplished with this technology [113].

Atrazine (ATR) is a triazine herbicide that is frequently used for controlling broadleaf weeds and perennial grasses in various crops in the US [114,115,116]. A novel electrochemical sensor was developed on a GCE that used MIP as a bioreceptor and was integrated with PtNPs and carbon nitride nanotube (C_3_N_4_ NT) nanocomposite for ATR detection. The sensor’s linearity range and LOD were calculated to be 1.0 × 10^−12^–1.0 × 10^−10^ and 1.5 × 10^−13^ M, respectively. The sensor showed high selectivity and sensitivity in wastewater samples compared to other analytical methods [73]. Glyphosate (GLY) is another highly used herbicide, and it is harmful to human wellbeing [117,118]. This study investigates the electrochemistry of GLY on a variety of electrode substances and electrolytes and provides quantitative information on GLY electroactivity at acidic, neutral, and basic pH levels on the surface of gold and platinum electrodes. The sensor’s performance was successfully moved to a microfluidic, chip-based platform. This made it possible to measure the concentration of tap water that had not been treated by using a disposable cartridge and vacuum filtration. The sensor’s low sample volumes, precise real-time monitoring, and low-cost components make it a promising solution for future decentralized evaluations of samples of drinking water, food, beverages, and biomedical samples [72]. Furthermore, a novel SPCE/AuNP@MIP was fabricated for GLY detection [119]. Malathion (MAL), an OP compound used as an insecticide, poses serious health and environmental problems [120]. A specific sensor was designed by modifying screen-printed gold electrodes (Au-SPEs) with MIP for MAL detection in olive oils and fruits. The morphology of the proposed sensor surface was studied using surface techniques, and performance characterization of the developed MIP sensor was conducted using electrochemical analysis techniques. Additionally, the sensor showed a wide concentration range that extended from 0.1 pg/mL to 1000 pg/mL, a low LOD of 0.06 pg/mL, and a recovery rate of 87.9%. The sensor was effectively used for MAL detection in real-time samples, promising new opportunities for detecting OP pesticide traces in various food items and environmental applications [74].

**Figure 7:**
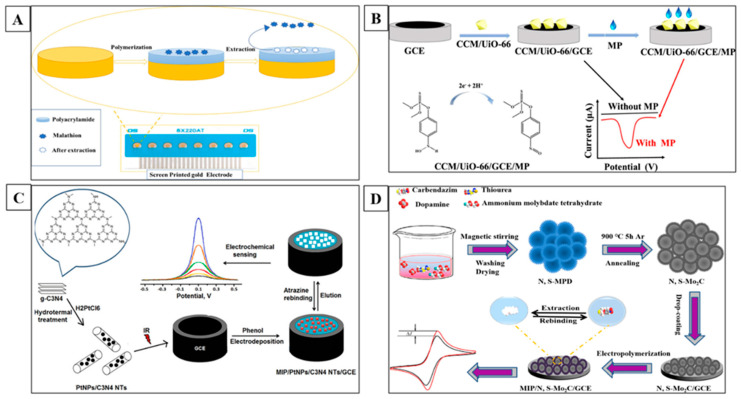
MIP-based electrochemical sensor for pesticide detection. (**A**) The Au-SPE-based MIP sensor is designed to detect MAL in olive oil and fruits [74]. (**B**) MOF-based MIP sensor for the detection of MP [113]. (**C**) A GCE MIP-based nanosensor for ATR detection [73]. (**D**) Detection of CBZ using Mo_2_C/C spheres (N, S-Mo_2_C) integrated with MIP [75].

Carbendazim (CBZ) is a wide-range benzimidazole fungicide that is widely used in the agriculture sector to prevent disease growth in fruits and vegetables [121]. Its benzimidazole ring provides long-term stability, leading to its widespread distribution in water and soil environments, but it has been found to cause infertility and damage testicles in animals in research. As a result of the harmful effects that it has, the European Union and other countries have enacted stringent residue limits [122]. This study investigates the detection and quantification of CBZ. For this purpose, a film of MIP was fabricated on the surface of nitrogen- and sulfur-doped hollow Mo_2_C/C spheres (N, S-Mo_2_C). The sensor was prepared through a one-pot approach and carbonization at extreme temperatures. The N, S, and Mo_2_C were characterized using various techniques, including scanning electron microscopy, Fourier-transform infrared spectroscopy, TEM, and CV. The sensor’s practicality and reliability were demonstrated when it was applied to detect CBZ residue in fruits and vegetables, indicating its potential in various fields such as food quality control, drug monitoring, drug quality control, and environmental monitoring [75]. Additionally, the HKUST-1@MIPs-GE electrode was developed for CBZ detection [123]. Figure 7 represents the schematic illustrations of some of the important MIP based electrochemical sensors for the detection of pesticides.

In 2020, Wang. et.al developed a two-in-one electrochemical biosensor that measures pesticides and heavy metal ions using a dual-recognition aptazyme beacon (DRAB). The self-blocked DRAB with aptamer and DNAzyme was stimulated by pesticides, resulting in selective cleavage of a MB-tagged signal probe (SP) in the presence of metal ions. The released DRAB–pesticides complex can connect next to SP for cyclic cleavage, creating a signal-enhanced DNA nanomachine. An enhanced aptazyme with a changeable arm was produced by optimizing the 3′ terminal of DNAzyme, enabling versatile detection of food pollutants with specific aptamers. 

Using chlorpyrifos and Pb^2+^ as real samples, the group achieved great sensitivity, selectivity, and practicality in fresh fruit and vegetable samples [124]. A synchronized detection approach for numerous pesticides is urgently needed due to their cohabitation with vegetables and increased toxicity. Therefore, Huang and his coworker [125] developed electrochemical aptasensors where both Ag-Au and Cu_2_O-Au nanoparticles were synthesized and labeled with acetamiprid and MAL aptamers to create two new electroactive SPs. The base complementary pairing between aptamers and thiolated DNA oligonucleotide sequences hybridized the two probes on the electrode, creating a dual-signal electrochemical aptasensor for detecting acetamiprid and MAL on a modified GCE. The LOD for acetamiprid was 43.7 pg/mL, and for MAL it was 63.4 pg/mL. The aptasensor detected acetamiprid and MAL in spinach and grapes, and a good recovery rate was observed.

Since aptamers have so many advantageous properties, a large number of aptamer-based biosensor systems have, to date, been designed for the analysis of small molecules belonging to different classes. Although only a small number of aptamers have been chosen for pesticide detection, the potential of aptamer-based biosensors for pesticide detection is not fully exploited. Attention has recently been drawn to the detection of acetamiprid. Acetamiprid is a neurotoxic pesticide that is widely used. It belongs to the class of neonicotinoid pesticides, which are synthetic nicotine derivatives that function as agonists of the nicotinic acetylcholine receptor, paralyzing and eventually killing the contaminated organism. Due to its high toxicity, anyone who is exposed to contaminated environments may be in danger. Recently, acetamiprid detection has garnered attention. Acetamiprid, a neonicotinoid insecticide, causes paralysis and death in infected organisms by acting as a nicotinic acetylcholine receptor agonist, like nicotine. Its high toxicity puts people in polluted environments in danger [11,103,126]. Fei et al. overcame this obstacle by coating GO nanoribbons, CNTs, and AuNPs to create label-free aptasensors. They created an aptasensor for acetamiprid detection by using the resultant Au/MWCNT-rGONR composites as the support for aptamer immobilization. The proposed EIS-based aptasensor displayed a linear response for acetamiprid in the range from 5 × 10^−14^ M to 1 × 10^−5^ M, with an LOD of 1.7 × 10^−14^ M (S/N = 3) [103]. Chlorpyrifos is the name of another member of the pesticide family. It is one of the OP insecticides that is most frequently used in agriculture to reduce pests and increase yield. By utilizing the extremely high electron transfer capabilities of carbon black and GO@Fe_3_O_4_, Jiao and his team [127] created an incredibly sensitive aptasensor for the label-free electrochemical measurement of chlorpyrifos. They used chitosan due to its large specific surface area, optimal dispersibility, and superior electrical conductivity, which were employed to trap more GO@Fe_3_O_4_. With a large surface area from the GO and a uniform layer of Fe_3_O_4_ on top, the GO@Fe_3_O_4_ nanocomposite created a unique sensing film with strong synergistic effects. This allowed electron transfer to occur, enabling the sensitive detection of chlorpyrifos with a low LOD and excellent selectivity.

## 4. Conclusions and Future Scope

Recent developments in biomimetic nanosensing methodologies are critically described in this review. A special focus has been made toward MIP-based and aptamer-based sensing approaches for the detection of mycotoxins and pesticides. Critical challenges in the fabrication of biosensors, nanomaterial integrations, and real matrix analyses are elaborately discussed. Different modes of MIP-based sensing methodologies were discussed for important pesticide and herbicide detection. Individual advantages and disadvantages of the different label and label-free aptasensing strategies have been discussed.

Despite the significant progress made in detecting mycotoxins and pesticides in the last two decades, the early and accurate detection of infected crops, fields, and foods is still a challenge to be tackled. Electrochemical detection methods are emerging as an alternative rapid sensing methodology over conventional clinical screening methods. However, several challenges remain unsolvable and need to be addressed by the sensing scientific research community. Those challenges are: i. screening sample selection, preparation, and separation; ii. label and label-free biosensors; iii. fouling studies; iv. high sensitivity, selectivity, and reproducibility; v. portable/wireless electrochemical transducer systems; vi. multiplex sensing methodologies

The integration of nanomaterial-based electrode transducer surfaces with the biomimetic sensing assay has tremendously improved sensor performance in terms of sensitivity and selectivity. The development of nanomaterial-based electrochemical biosensors is now being pursued by researchers with the aim to dramatically enhance sensor sensitivity, selectivity, and repeatability when it comes to the measurement of toxicants. However, real sample matrix analysis is the biggest challenge in the development of electrochemical biosensors. The specific detection of toxins in agricultural and food products is a realistic challenge due to the complex structural matrix. Researchers are trying to simplify the real sample matrix medium by using ultrafiltration methods and the utilization of fouling coating layers to specifically allow smaller toxins molecules to reach the electrode surface area. There is still a lot of work that needs to be conducted in this area. One more challenge is the continuous monitoring of toxins in agricultural fields and products, food, and beverages/products. Wearable electrochemical sensing platforms such as tattoo sensors, microneedle sensors, and textile-based sensors would help in gaining access to the toxins content in medium real-time conditions, and the integration of flexible portable potentiostats with biosensing assays could enhance the continuous monitoring modality.

## Figures and Tables

**Table 1:** Summary of electrochemical MIP-based sensors for the detection of mycotoxins and pesticides.

Analyte	Electrode Interface	Detection Range	Limit of Detection	Sample	Reference
MYCOTOXINS					
AFB1/FuB1	ITO/PANI-MIP-AFB1 and FuB1	1 pg/mL to 500 ng/mL	0.313 and 0.322 pg/mL for AFB1 and FuB1	corn extract	[41]
Patulin	MIP-capped AgNPs@ZnMOF/patulin	0.1–10 μmol/L	0.06 μmol/L	water and apple juice	[58]
AFM1	AuNP/allay mercaptan/plasmonic chip/MIP film/AFM1	0.0003–20.0 ng/mL	0.4 pg/mL	milk	[55]
OTA	GCE-MWCNT-Nafion-Ru(bpy)_3_	10 fg/mL to 10 pg/mL	0.03 ng/mL	corn	[67]
OTA	GCE-MWCNT-MIP	0.050 and 1.0 µM	1.7 µg/L	beer and wine	[43]
OTA	GCE-Ru Se NPs-MIP	0.001 to 100 ng/mL	0.2 pg/mL	milk and peanut Oil	[68]
FuB1	GCE-AuNPs-Ru@SiO_2_ NPs-MIP	0.005–5 ng/mL	0.35 pg/mL	seafood	[69]
AFB1	Au electrode-PATP-MOF	3.2 fM and 3.2 µM	3 fM	spiked rice samples	[70]
**PESTICIDES**					
Diazinon	MOF/MIP/NTD/Dia	0.002–0.03 mg/m^3^	0.02 mg/m^3^	air	[71]
GLY	SPCE/AuNP/MIP/Gly	273–1200 pg/mL	0.8 pg/mL (DPV) and 0.001 pg/mL (EIS)	agri-food sample	[72]
ATR	Pt NPs/C_3_N_4_NTs/MIP/ATR	1.0 × 10^−12^–1.0 × 10^−10^ M	1.5 × 10^−13^ M	wastewater	[73]
MAL	Au-SPE/MIP/MAL	0.1–1000 pg/mL	0.06 pg/mL	olive fruits and oils	[74]
CBD	N, S–Mo_2_C/MIP/CBD	1 × 10^−12^∼8 × 10^−9^ M	6.7 × 10^−13^ M	fruits and vegetables	[75]
Paraquat	SPCE-PtNPs@SiO_2_-vinyl NPs	0.05 to 1000 μM	0.02 nM	vegetable samples	[76]
Carbofuran	Fe_3_O_4_@Au-MIP-NH_2_/GCE	0.01 to 100 mM	1.7 nM	fruits and vegetables	[77]
Dinotefuran	GCE/MIP/PVC	10^−7^ to 10^−2^ M	0.35 mg/L	cucumber	[78]

MIP: molecular imprinted polymer; OTA: ochratoxin A; AFB 1: aflatoxin B 1; FuB1: fumonisin B1; ATZ: atrazine; MAL: malathion; CBD: cannabidiol, GLY: glyphosate; SPCE: screen-printed carbon electrode.

**Table 2:** Summary of electrochemical aptasensors for the detection of mycotoxins.

Mycotoxins	Electrode Interface	Detection Range	Limit of Detection	Sample	Reference
OTA	MB and thiol dual-labelled modified gold electrode with aptamer	0.1–1000 pg/mL	0.095 pg/mL	red wine	[79]
OTA	On the ITO electrode surface, MB-labelled electroactive mononucleotide diffuses.	0.01–1.0 ng/mL	0.004 ng/mL	oats	[80]
OTA	Au electrode is used to immobilize rolling circle amplification products.		0.065 pg/mL	red wine	[81]
OTA	Ag NPs/Au electrode	0.3–30 nM	0.05 nM	beer	[82]
OTA	AuNPs with DNA functionalization were fixed on the electrode.	2.5 pM–2.5 nM	0.75 ± 0.12 pM	red wine	[83]
OTA	AuNPs altered tags: N-(4-aminobutyl)-N-ethylisoluminol; Au electrode	0.02–3.0 ng/mL	0.007 ng/mL	wheat	[84]
AFB1	GO-based aptamer	0.05–6.0 ng/mL	0.05 ng/mL	milk	[20]
AFM1	Pt microelectrode-Fe_3_O_4_ NPs-PANI-APT	6–60 ng/L	1.98 ng/L	milk	[85]
AFB1	SPCE-FGO-HMDA-MB-APT	006 and 0.02 ng/mL	0.05 ng/mL	milk	[86]
OTA	CFME-Ag NPs-APT	0.07 to 10 nM	0.05 nM		[87]
OTA	GCE-mesoporous silica NPs-APT		0.003 nM	wheat	[88]
OTA	Nitrogen-doped GR QDs-SiO_2_-APT	10 fg/mL to 10 pg/mL	0.5 pg/mL	corn	[67]
OTA	AuE-rGO-Au NPs-APT	1 pg/mL to 50 ng/mL	0.3 pg/mL	red wine	[89]

GCE: glassy carbon electrode; AuE: gold electrode; rGO: reduced graphene oxide; AuNPs: gold nanoparticles; AgNPs: silver; NaMB: methylene blue; PANI: polyaniline; APT: aptamer.

## Data Availability

Not applicable.
